# Differential effects and success stories of distance education in Covid-19 lockdowns on the development of reading comprehension in primary schools

**DOI:** 10.1007/s11145-022-10369-0

**Published:** 2022-10-21

**Authors:** Eliane Segers, M. In ’t Zandt, J. Stoep, L. Daniels, J. Roelofs, J. Gubbels

**Affiliations:** 1 Expertisecentrum Nederlands (Dutch Center for Language Education), Nijmegen, The Netherlands; 2 Educational Sciences, Radboud University, Nijmegen, The Netherlands; 3 KBA Nijmegen, Nijmegen, The Netherlands; 4 Behavioural Science Institute, Radboud University, Thomas van Aquinostraat 4, 6525 GD Nijmegen, The Netherlands

**Keywords:** Covid-19, Reading comprehension, Primary school, Lockdown, SES, Parent involvement

## Abstract

In the current study, the development in reading comprehension performance of students in lower-SES versus higher-SES schools during and after school closures due to Covid-19 lockdowns was examined, and compared to a normed reference group. Furthermore, we explored protective factors against negative effects at the time of school closures, by pinpointing successful practices in a sub sample of resilient lower-SES schools. The total sample consisted of 2202 students followed from grade 2–4. Overall, we found that students in lower-SES schools made less progress over time than students in higher-SES schools. On average, students made less progress during the lockdowns, but here, the interaction with SES was not significant. Students' reading comprehension levels partially recovered after the lockdowns. Questionnaire-data revealed that schools were better prepared during the second lockdown, with teachers making more use of digital means, and providing more online reading instruction. In addition, collaboration with the parents seemed to have improved. The in depth interviews with resilient lower-SES schools revealed that the introduction of online education and investing in educational partnerships with parents may have helped to minimize the negative impact of lockdowns. We conclude that lockdowns have a negative effect on the development of reading education, but that students are resilient. Digital means and partnership with parents may be seen as protective factors to attenuate the negative effects of emergency remote teaching.

## Introduction

In the current knowledge society, reading comprehension is a crucial skill in which students from low socio-economic or migrant backgrounds often lag behind (Melby-Lervåg & Lervåg, 2014). School closures and emergency remote teaching (Dachsler et al., 2021) as a consequence of the Covid-19 pandemic may have enlarged this gap (Hammerstein et al., 2021), but it is not clear to what extent children's reading comprehension levels have recovered after the lockdowns. Schools differed greatly in their setup and quality of this distance education, and it is important to document success stories, so schools can be better prepared for the future. In the current study, we combined quantitative and qualitative information to examine the impact of two Covid-19 pandemic lockdowns in the Netherlands and understand variation in this impact. The first aim was to investigate whether the gap in reading comprehension performance of schools with students from low socio-economic or migrant backgrounds (see e.g., Ransdell, 2012) versus schools with students from high socio-economic backgrounds increased over de periods of school closure due to Covid-19 and to what extent schools would recover afterwards. The second aim was to explore protective factors against such effects, by pinpointing successful practices of resilient schools at the time of school closures.

### Impact of school-SES on reading comprehension

Environmental factors (often assessed via measures of socio-economic status, SES) have been shown to play an important role in the development of reading comprehension, both on the individual level as well as at school level. For example, Ransdell (2012) showed that reading comprehension is highly related to SES at school level. In an influential study across high schools in the USA, Caldas and Bankston (1997) showed that the SES level of classmates impacts general school achievement over and above individual SES level. This result has been replicated in many studies across the globe, for example, by Tse and Xiao (2014) in the PIRLS 2014 dataset of fourth graders in Hong Kong: higher school-SES was related to higher reading comprehension scores, after controlling for individual SES (see also Chiu & McBride-Chang, 2006; Perry & McConney, 2010).

In line with this result, Hart et al. (2013), studying gene*environment interactions, showed a main effect of school-level SES on reading comprehension in a large sample of third- and fourth grade pupils, independent of family-level SES. They embedded this latter result in the bioecological model of Bronfenbrenner and Ceci (1994), suggesting that students in low-SES schools are less likely to develop their full reading potential, as the role of genetic influences is smaller in such environments. Kieffer (2012) showed that school-SES had a strong impact on reading growth between third and eighth grade, with pupils in schools with a lower SES showing slower reading growth. He suggested that the effect of the environment may play a greater role in processes involved in advanced reading comprehension, such as vocabulary and oral language comprehension (as opposed to decoding, see e.g., Verhoeven & Van Leeuwe, 2008).

### Impact of school closures on reading comprehension

School closures have an impact on reading comprehension. Both the effect of school closures with no education during closure (i.e., a holiday) and the effect of school closure with education during closure (as during pandemic lockdowns) have been studied. The first set of studies gives an indication of the differential impact that school closure may have on various populations, and while a summer holiday is very different from a lockdown, it is an indication of the effect of school closures, albeit that there is not distance education.

A narrative and meta-analytic review by Cooper et al. (1996) indicated an overall negative effect of summer vacation on reading comprehension. The effect was larger in higher grades, and especially lower-SES students (based on income level) tended to decline. Alexander et al. (2007) retrospectively studied the achievement gap of high-SES versus low-SES 9th graders and showed that the summer shortfall had a cumulative negative effect on low-SES students, starting in the early school years. Several more recent studies have also looked at SES at the school level. Tiruchittampalam et al. (2018) found that students from lower-SES schools tend to lose gains made during the school year over the summer holiday, while students from higher-SES schools made gains for oral passage reading but not for reading comprehension. The authors contributed the latter lack of effect to low sensitivity of the test. In an attempt to explain such differential effects, Entwisle et al. (2001) coined the term “faucet theory”. During the school year, resources are available for all. During the holidays, higher-SES families provide resources that attenuate the effect of not being in school. Such resources are not just materials but are also present in parental expectations and activities (see Sénéchal & LeFevre, 2014). For children from lower-SES homes, these compensatory resources often are absent (Leefatt, 2015).

Overall, it is clear that a school closure during the summer holidays has a stronger negative effect for students attending low-SES schools. After the Covid-19 lockdowns, several studies examined whether this effect would also hold during school closures with emergency remote teaching. Hammerstein et al. (2021) published a first systematic overview of research regarding the effects of Covid-19 school closures that included nine studies. Overall, they reported a negative effect of these school closures, comparable to those of summer holiday closings, which especially affected students from families with a low SES. Indeed, Engzell et al. (2021) reported an average learning loss of about one-fifth of a school year, but losses were much higher for low-SES students after the first 8-week lockdown for primary schools in the Netherlands. Haelermans et al. (2022) reported on the same lockdown period in Dutch primary schools. Overall, they showed an average delay of 5.5 weeks for reading, and an enlargement of existing inequality. The negative effects were larger for students with low-educated parents or parents with a lower income. Migration background did not have an additional negative effect. Similar results were found in Belgium (Flanders region) where primary schools were closed for 9 weeks (including two holiday weeks). Maldonado and De Witte (2022) found larger learning losses in reading comprehension for schools with a low-SES population and attributed this to lower levels of home-schooling by the parents (in line with the faucet theory). Schult et al. (2022) studied 5th graders in Germany and found slightly lower scores in reading comprehension after a 2-month lockdown in 2020 (− 0.07 SD), with school characteristics playing a minor role. Crosson and Silverman (2022) had fifty K-2 USA public school teachers fill out questionnaires. Schools in the USA were not fully closed during the pandemic, but on average experienced 192 days of partial closure (Unicef, 2021). The teachers indicated that they, overall, implemented less literacy instruction, especially regarding vocabulary and reading comprehension strategies. In summary, converging evidence shows learning losses during lockdowns, especially for students in low-SES schools. Possible recovery effects have not been studied so far. In addition, there is a lack of research on what schools have done to prevent negative effects.

### Current study

Engzell et al. (2021) noted the Netherlands to represent a “best-case scenario due to the country’s short school closures, high degree of technological preparedness, and equitable school funding” (p.5), but still found that students’ learning stagnated during the lockdown. They argued that research would be needed to find out whether students recover from the lockdown. In addition, it remains unclear whether, in the case of a second lockdown the Netherlands experienced in the winter of 2021–2022, schools would have learned from the first lockdown, making the effects less strong. However, Hammerstein et al. (2021) suggested additional losses, and argued that insight is needed in potential compensatory measures. Indeed, the review by Zierer (2021) concluded that there was a high heterogeneity in effects of school closures, and called for research to study school organizational, pedagogical and didactic concepts that would help minimize the effects. Since especially schools with low-SES populations seem to be affected by lockdowns, it is relevant to find out which characteristics can be distinguished in low-SES schools that were resilient for the negative effects of lockdowns.

In the current study, we thus asked the following research questions:

1. What is the impact of Covid-19 lockdowns on the development of reading comprehension and to what extent does school-level SES moderate this effect?
2. How did schools design their distance education during Covid-19 lockdowns?
3. What are the characteristics of resilient low-SES schools?

We expected a negative impact of the Covid-19 lockdowns on the development of reading comprehension, especially in lower SES-schools, and a recovery after the lockdowns, especially in the higher-SES schools. Second, we expected schools to be better prepared for distance education during the second lockdown. Finally, we expected resilient lower-SES schools to have a good digital climate, with educational partnership between parents and teachers, and an ongoing monitoring on the development of the students.

## Method

### Research design

The present study is a sub-study of a large-scale assessment (PIRLS 2021) of reading comprehension of Dutch primary school students in which 111 schools took part. For the current study, these schools were invited to participate via a letter that explained the aim and procedure of the study.

**Fig. 1:**
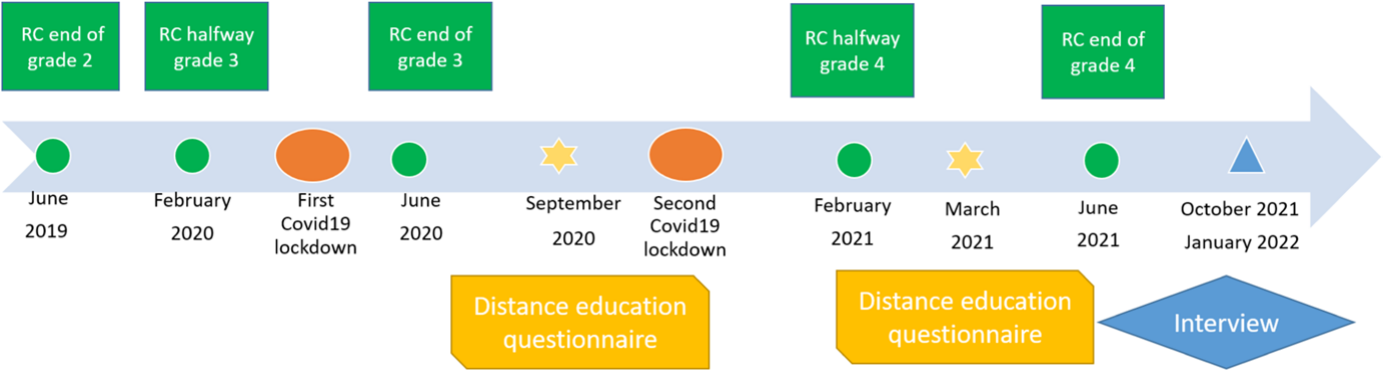
Visual representation of the research design. *RC* reading comprehension. Icons from the Noun Project: *Classroom* by Adrien Coquet, *Communication* by Eucalyp, *Covid* by Dimas Nanda, *Critical path method* by M. Oki Orlando, *Difficult* by Webtechops LLP, *Distance education* by Riyan Resdian, *Find* by I Putu Kharisn, *Handshake* by Eko Purnomo, *Homework help parent* by Gan Khoon Lay. https://thenounproject.com/

The current study applied a mixed method design in which test and questionnaire data was combined with interview data. Figure 1 provides an overview of the timing of the various research methods that were used in this study with reading comprehension (RC) assessments indicated in green, questionnaires in yellow, and the interview in blue. During this time, schools experienced two lockdowns. The first lockdown was between March 15th 2020 and June 8th 2020. Until May 11th (i.e., 8 weeks, including a 2-week holiday) schools were closed, from May 11th till June 8th (i.e., 4 weeks), students attended schools 50% of the time. The second lockdown was from December 14th 2020 till February 8th 2021 (i.e., 8 weeks including 2 weeks of Christmas holiday). This is a total of 60 days fully closed and 20 days partially closed, which is high compared to other European and Central or East Asian countries (Unicef, 2021).

Primary schools in the Netherlands are required to track pupil’s progress via a student monitoring system. This way, all schools monitor reading comprehension levels of the children twice a year, using standardized test that are included in this student monitoring system. In order to gain insight into the impact of Covid-19 lockdowns on the development or reading comprehension, we gathered students’ reading comprehension scores from an independent reading comprehension assessment (i.e., Cito Leerlingvolgsysteem, 2016). This assessment is used by most Dutch schools and consists of an assessment halfway the schoolyear (i.e., February) and at the end of the schoolyear (i.e., June). For the present study, we asked schools to provide the reading comprehension scores of the two measurements before the first lockdown (i.e., June 2019 and February 2020), the measurement after the first lockdown and before the second lockdown (i.e., June 2020), and the two measurements after the second lockdown (i.e., February 2021 and June 2021). Together, these scores show students’ reading comprehension development from the end of 2nd grade to the end of 4th grade.

The reading comprehension scores of the groups in our sample were compared to a reference group (percentile 25–75), data of which is available from the student monitoring system. The percentiles of the reference group are based on the scores of a large representative sample of Dutch students who finished at least three consecutive assessments of the reading comprehension test (for example end 2nd grade, halfway 3rd grade and end 3rd grade). Sizes of the samples of the individual assessments that we use in this study (from end of 2nd grade to the end of 4th grade), ranged from 1466 to 2421 students. In addition, we asked school principals to complete a questionnaire about their design of distance education during the Covid-19 lockdowns. School principals were invited in fall 2020 to complete the questionnaire that addressed their school practices during the first lockdown. Since there was a second lockdown in the winter of 2020/2021, an adapted version of the questionnaire was sent to all participating school principals in spring 2021 to gain insight into their educational practices during this second lockdown.

As an indicator of the vulnerability of the student population of a school, Dutch schools are assigned a ‘school weight’ by the Dutch government (CBS, 2019). The school weight of a school is based on the educational level and country of origin of the parents of all students, the average education level and duration of stay of the mother of all students, and the proportion of parents that have debt restructuring arrangements. School weights range from 20 to 40 following a normal distribution with a mean of 30. The higher a school’s weight, the lower the school’s SES and the more vulnerable the student population of that school. In the present paper, schools with a weight lower or equal to 26.5 (25% of the schools) were considered to be high-SES schools, schools with a weight between 26.6 and 30.9 (50% of the schools) were classified as medium-SES schools, and schools with a weight higher or equal to 31.0 (25% of the schools) fell in the category of low-SES schools. Interviews with resilient low-SES schools were held from October 2021 to January 2022 to gather in-depth information about their educational practices during both Covid-19 lockdowns and the restart of education, after the lockdowns.

### Participants

For the present study, 76 of the schools in our sample delivered data on the reading comprehension scores of their students for the five different measurements. This resulted in data of 2539 students, but some of these students were excluded due to the following reasons: (1) student had one or more score(s) that were not possible, probably due to a mistake in data entry by the schools (*N* = 2), (2) student was administered a different test than the Cito test at one or more measurements (*N* = 156), and (3) student had less than three scores on the five measurements (*N* = 179). After excluding participants who did not meet the inclusion criteria, the reading comprehension scores of 2202 students from 69 different schools remained and were examined. Of these 2202 students, 49.2% were male. The average age of the students on June 30th, 2019, was 8 years and 97.08 days (SD = 167.35 days). The average school-level SES of these school in 2020 was 28.80 (*SD* = 3.89).

The questionnaire about the design of distance education during Covid-19 lockdowns was completed by a subsample of 35 schools for the first lockdown and 64 schools for the second lockdown. Some schools (n = 23) completed both questionnaires. These schools were included in both the description of practices during the first Covid-19 lockdown and the description of practices during the second Covid-19 lockdown.

For the in-depth interviews, the schools that were approached for the interview met the following three criteria: (1) a school weight ≥ 31 (low SES schools), (2) grade 4 students achieved above-average scores at two or more measurements periods, and/or showed above-average growth in the period June 2019–June 2020 and/or June 2020–June 2021, based on the reading comprehension data (note: the average of each measurement moment was based on the scores of all low-SES schools), and (3) the distance education questionnaire had been completed by the school leader. Seven primary schools that met the criteria were approached by email, five of which eventually participated in the interviews.

### Materials

#### Reading comprehension test

Reading comprehension was assessed via a standardized test (Cito, 2016). The test consists of a number of texts that vary in text type and genre. Students first read (part of) the text and then answer multiple choice questions about the text. The questions provide insight in four types of reading comprehension processes: understanding, interpreting, looking up, and summarizing information. An example of a question is ‘What will this text be about?’. The test is divided over three tasks that each comprise a total of 24 or 25 items that belong to multiple texts. All items can be placed on the same IRT reading comprehension scale based on the level of difficulty of the item. Based on students’ responses to the various items, a reading comprehension skill score is calculated (ranging from zero to 388).

#### Distance education questionnaire

A questionnaire was developed to explore school practices during Covid-19 lockdowns based on existing questionnaires of the Dutch Inspectorate of Education (2020) and the Monitor Hybrid Education (Smeets, 2020). The questionnaire consisted of 32 closed items divided over four main topics. The first theme concerned the general design of distance education during Covid-19 lockdowns and the (partial) restart of education after the lockdown, which was assessed with five items. Next, school principals answered three items about the actions they undertook to reach students in vulnerable home situations and success factors and difficulties that they experienced during the lockdowns. All items for these first two topics were checkbox questions. The third topic that was addressed in the questionnaire was the design of reading education during the lockdowns. This topic comprised fourteen statements describing teacher practices in reading education. Topic four concerned monitoring of reading achievements and consisted of ten items. For both teacher practices in reading education and monitoring of reading achievement, school principals indicated on a 5-point Likert scale (*1* = *never; 2* = *occasionally (1–25% of the time); 3* = *regularly (25–50% of the time); 4* = *often (50–75% of the time); 5* = *very often (*> *75% of the time)*) how often these practices occurred in their schools. The principals answered this question for three time periods: before, during, and after the lockdown (during the restart). In the questionnaire about the first Covid-19 lockdown, teachers also indicated their teachers’ practices at the start of the new school year.

We focused on three themes: digitalization, monitoring of reading achievement, and contact/educational partnership with parents. Based on the content of the items, four items about teachers practices in their design of reading education were used to gain insight in digitalization and four items for monitoring of reading achievement. For both themes, only the answers that referred to the period of the Covid-19 lockdown were used. As an indicator for the contact with parents, two items of the questionnaire were used. The first item was ‘Looking back on the period of the Covid-19 lockdown, what were prominent/positive/powerful aspects that characterize your school?’. One of the checkbox options for this item was ‘the collaboration with parents/caretakers’. This item was included as a dichotomous variable based on school principals answer whether this was a prominent, positive, or powerful aspect that characterized their school during the Covid-19 lockdown (*1* = *yes; 2* = *no*). The second item read ‘Which specific difficulties did you experience during the period of the Covid19 lockdown?’. The checkbox option ‘parents were less available or inclined to support the student during the moments that their child worked on schoolwork at home’ was included as a dichotomous variable as well (*1* = *yes; 2* = *no*).

#### Interviews

Based on the distance education questionnaire, a semi structured interview guide was developed, including the following topics:

- form and content of reading instruction during Covid19 lockdown and (partial) restart of education,
- the balance between homework and schoolwork in reading during the (partial) restart of education,
- communication with students and parents about reading instruction and reading achievement for the overall period,
- educational partnership with parents regarding reading for the overall period,
- monitoring reading outcomes during the overall period.

### Procedure

The reading comprehension test was administered by the schools twice a year. The first test took place in the period between halfway January and halfway February, the second test in June of each school year. The teacher administered the test to the group of students, preferably one task a day (Cito, 2016). One task took the students about 45 min and in total students completed three tasks. It is allowed to administer two tasks in 1 day, as long as students get a proper break in between. All participating schools were asked to send the test data of the five measurements (see Fig. 1) to the researchers in standardized formats via mSafe. Schools used unique codes to identify students, so that anonymity of the students could be guaranteed. Parents received an information letter that explained the aim and procedure of the study. Parents that did not want their child to participate in the study could withdraw their permission to collect and process data until 3 months after the study was completed. Schools that delivered the data received a €50-giftcard as compensation for their effort.

The questionnaires were completed online via Limesurvey by school principals of the participating schools. School received an email with a unique login code for the first distance education questionnaire in December 2020. After a few weeks, a reminder to complete the questionnaires was sent. In March 2021, the invitation with the unique login code for the second distance education questionnaire was sent to all principals, with a reminder in April 2021. It took school principals about 10 min to complete the first questionnaire and about 15 min to complete the second questionnaire.

Interviews were administered by one of the authors and recorded via videoconferencing tools (Zoom or Teams) and lasted approximately 60 min. Schools that participated in the interview received additional financial compensation and a school report. The interviews were anonymously transcribed and coded by one of the authors and an assistant in ATLAS.ti Mac (Version 9.1.3).

### Data-analysis

To answer the first research question, concerning the development of reading comprehension scores and the effect of school-level SES on this development, the student-level reading comprehension scores were used. The data preparation and analyses were done with R (R Core Team, 2021).

The student-level data contained several missing values in the outcome variable and the control variables; therefore, the data were imputed using the *mice* function of the *mice* package (Van Buuren & Groothuis-Oudshoorn, 2011) before executing the analyses. The imputation method *2l.pmm* from the *miceadds* package (Robitzsch & Grund, 2021) was used to impute the data and five imputations were run. Several warnings occurred due to the varying ranges of the variables, therefore the continuous variables were scaled and centered before proceeding with the analyses.

Before running the analyses, some descriptive statistics and correlations were obtained. The pooled means and standard deviations were computed using the *with* function of the *mice* package (Van Buuren & Groothuis-Oudshoorn, 2011), and the pooled correlations were obtained using the *micombine.cor* function of the *miceadds* package (Robitzsch & Grund, 2021).

For the analyses of students’ reading comprehension development, multiple mixed models were tested before the final model was created. The first model contained reading comprehension score as an outcome variable and fixed effects for time, school-level SES, the interaction between time and school-level SES, as well as age and gender as control variables. The random effect of time varying over students and the random effects of time, school-level SES, and the interaction between time and school-level SES varying over classes (including the fixed and random intercepts) were also specified in the model. An error appeared because the number of observations was insufficient to run this model. Therefore, the model was simplified by first removing the random correlations, next the random effect of time varying over students and the random effect of school-level SES varying over classes, and finally also the random interaction effect of time and school-level SES varying over classes. The final model consisted of reading comprehension score as outcome variable, with fixed effects of time, school-level SES, the interaction between time and school-level SES, age and gender as control variables, and a random effect of time varying over classes. The fixed and random intercepts, from the student- and class level, were also included in the model. This final model was fitted using the *with* function of the *mice* package (Van Buuren & Groothuis-Oudshoorn, 2011).

The multicollinearity of the predictors in the model was investigated by running the model on the five imputations of the data separately with the *with* function of the *mice* package (Van Buuren & Groothuis-Oudshoorn, 2011). With the *vif* function of the *car* package (Fox & Weisberg, 2019) the VIF and GVIF values for the effects were obtained for the five different imputations. The GVIF values of the effects including factor variables, the effect of time and the interaction, were squared in order to compare this value with the VIF threshold. The GVIF of school-level SES, a continuous variable, was equal to the VIF value. These GVIF^2^/VIF values of the effects were all below the VIF threshold of 2.5 which indicates considerable collinearity (Johnston et al., 2018), for all five imputations. Therefore, we assumed multicollinearity was not a problem for the analyses.

The final pooled parameter estimates were obtained using the *testEstimates* function of the *mitml* package (Grund et al., 2021). To obtain all pairwise comparisons for the different measurements, the reference category of time was adjusted four times using the *relevel* function of the *stats* package (R Core Team, 2021). The model was run five times, each time with a different measurement as reference category. However, due to the categorical variable time (values ranging from 1 to 5), the five different models contained dummy variables for time and the interaction effect. Therefore no overall main effect of time, nor an overall interaction effect between time and school-level SES could be derived from the five models. In order to obtain the overall main effect of time and the overall interaction effect between time and school-level SES two separate Wald tests were performed using the *D1* function of the *mice* package (Van Buuren & Groothuis-Oudshoorn, 2011). The Wald tests compared a full model and a reduced model to examine whether the difference between the two models, namely the effect of interest, led to a significantly better fit of the model. For the first Wald test the effect of interest was the main effect of time and for the second Wald test this was the interaction between time and school-level SES.

In order to visually display the results in an understandable way, the students were divided into three groups based on the school weight of their school: low school-level SES (values ≤ 26.5), medium school-level SES (values between 26.6 and 30.9), and high school-level SES (values ≥ 31.0). The reading comprehension scores of the students in these three groups were plotted against the scores of the reading comprehension test of the reference group. These scores contain three bounds and the scores in between this range (percentile 25–50 and 50–75). In addition to the visualization, several t-tests were performed using SPSS version 20 (IBM Corp, 2011) to test whether the reading comprehension scores of the students was different than those of the reference group at the 50th percentile.

## Results

### Descriptive statistics

The development of the average reading comprehension scores of all 2202 students is shown in Table 1. In addition, the correlations between students’ reading comprehension scores and school-level SES for the five measurements are presented in Table 1.

**Table 1:** Means and standard deviations of reading comprehension scores over five measurements and correlation with school-level SES

	June 2019	Feb 2020	June 2020	Feb 2021	June 2021
*Reading comprehension score*					
Mean	145.20	156.39	158.91	169.31	178.66
Standard deviation	27.60	27.06	28.59	27.01	28.71
Correlation with school-level SES (*Pearson r*)	0.15*	0.22*	0.21*	0.27*	0.28*

^*^p < 0.001

### Development of reading comprehension during lockdowns

We first examined the development of reading comprehension within the current sample. The mixed model analysis of students’ reading comprehension scores from before the first Covid-19 lockdown to past the second lockdown showed a significant interaction effect between time and school-level SES (*p* = 0.001), as presented in Table 2, showing the overall main effect of time and interaction effect between time and school-level SES. This indicates that the development of the reading comprehension scores over the five measurements differs for different values of school-level SES. Because of the significant interaction effect between time and school-level SES, the two main effects were not further investigated. We did include the test statistics for the main effect of time in Table 2, while the test statistics of the main effect of school-level SES for the five different models can be found in Table 3. These statistics show that also the main effects of time and school-level SES were significant.

**Table 2:** Test statistics for the interaction effect between time and school-level SES on reading comprehension scores

	*F*	*df1*	*df2*	*dfcom*	*p*
*Effect*					
Time	142.21	8	1202.08	10,981	< 0.001
Time × school-level SES	4.56	4	1073.48	10,984	0.001

**Table 3:** Test statistics for the main effect of school-level SES on reading comprehension scores in the five different models

	*b*	*SD*	*t*	*df*	*p*
*Main effect of school-level SES*					
Model 1	0.10	0.04	2.68	56.24	0.010
Model 2	0.18	0.03	5.33	139.39	< 0.001
Model 3	0.17	0.03	5.68	485.66	< 0.001
Model 4	0.22	0.03	7.16	77.97	< 0.001
Model 5	0.25	0.03	7.43	136.53	< 0.001

In Model 1 the reference category is measurement 1, in Model 2 it is measurement 2, etc.

**Fig. 2:**
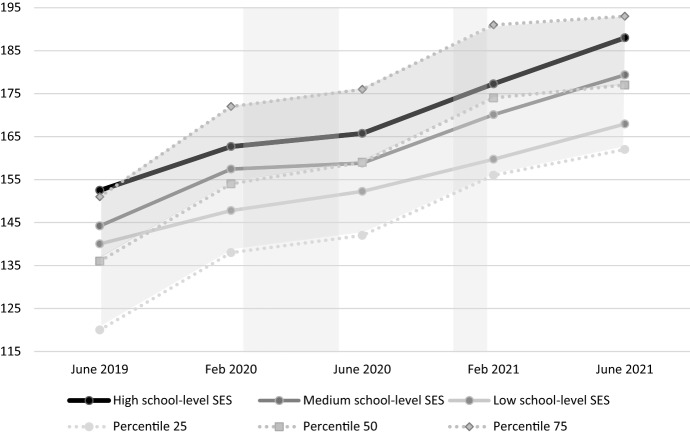
Development of the average reading comprehension scores between June 2019 and June 2021, for students with high school-level SES, medium school-level SES, and low school-level SES, in comparison to the reference group. The reference group is represented by the two curved grey areas, corresponding to percentiles 25–50 and 50–75. the two rectangular grey areas represent the first and second lockdown period

To further investigate the effect of school level-SES on the development of reading comprehension scores during the two lockdowns (the interaction effect), the development between the second and third measurement (period of first lockdown) and the third and fourth measurement (period of second lockdown) was analysed in more detail. School-level SES had no significant effect on the development of reading comprehension scores during the first (*p* = 0.860), nor during the second lockdown (*p* = 0.152). Further analyses showed that the effect of school-level SES on the development of reading comprehension scores was significant between measurement 1 and 2 (*p* = 0.046), measurement 1 and 4 (*p* = 0.002), measurement 1 and 5 (*p* < 0.001), measurement 2 and 5 (*p* = 0.015), and measurement 3 and 5 (*p* = 0.016). In Fig. 2, these interaction effects are visualized. Here, we also included results from the reference group. We can observe that students at lower school-level SES schools started above the 50th percentile score of the reference group for reading comprehension before Covid, but about 5 months after the second lockdown, these students’ scores were close to the 25th percentile of the reference group. Furthermore, students at medium-level schools started between the 50th and the 75th percentile, and ended close to the 50th percentile.

We next compared the development of reading comprehension of the full current sample to that of the reference group (that is not differentiated on SES) at the 50th percentile. Before the lockdowns, at measurements 1 and 2, the current sample had higher scores than the reference group (M_current1_ = 145.2, SD = 26.394, M_reference1_ = 136, *p* < 0.001; M_current2_ = 156.4, SD = 26.645, M_reference2_ = 154, *p* < 0.001). After the first lockdown, at measurement 3, the scores did not differ (M_current3_ = 158.9, SD = 25.512, M_reference3_ = 159, *p* = 0.867), while after the second lockdown, the current group scored below the 50th percentile of the reference group (M_current4_ = 169.3, SD = 24.843, M_reference4_ = 174, *p* < 0.001). A recovery effect was found in the months after the lockdowns, as the current sample scored again a bit higher than the reference group at measurement 5 (M_current5_ = 178.7, SD = 27.848, M_reference5_ = 177, *p* = 0.005). While effect sizes are small, the difference in growth for the reference group versus the current sample between measurement 2 and 4 (from February 2020 to February 2021) are substantial. The current sample had 35.5% less growth (12.9 vs. 20), which could be extrapolated to a delay of 4.2 months. Overall, thus, the results show that the lockdowns had a negative effect on reading comprehension development across school-level SES.

### Design of distance education during Covid-19 lockdowns

The answers on the questionnaires provided insight in the way schools designed their distance education during the Covid-19 lockdowns about digitalization, monitoring of reading achievement, and contact with parents. Table 4 shows the results regarding digitalization during the first lockdown as well as during the second lockdown.

**Table 4:** Teachers’ digitalization practices during Covid19 lockdowns

	First Covid19 lockdown (March 2020–May 2020) n schools = 35					Second Covid19 lockdown (December 2020–February 2021) n school = 64				
	n (%) never	n (%) occasionally	n (%) regularly	n (%) often	n (%) very often	n (%) never	n (%) occasionally	n (%) regularly	n (%) often	n (%) very often
*Digitalization*										
Teachers use a digital environment to collaborate and assign tasks	10 (28.6%)	4 (11.4%)	4 (11.4%)	6 (17.1%)	11 (31.4%)	8 (12.5)	6 (9.4%)	16 (25.0%)	9 (14.1%)	25 (39.1%)
Teachers provide online reading instruction	7 (20.0%)	8 (22.9%)	9 (25.7%)	7 (20.0%)	4 (11.4%)	6 (9.4)%	7 (10.9%)	12 (18.8%)	15 (23.4%)	24 (37.5%)
Teachers use digital material from the coursebook for reading	6 (17.1%)	6 (17.1%)	5 (14.3%)	7 (20.0%)	11 (31.4%)	14 (21.9%)	6 (9.4%)	11 (17.2%)	11 (17.2%)	22 (34.4%)
Students use digital software to practice reading	10 (28.6%)	4 (11.4%)	5 (14.3%)	5 (14.3%)	11 (31.4%)	15 (23.4%)	9 (14.1%)	11 (17.2%)	10 (15.6%)	19 (29.7%)

Never = 0% of the time; occasionally = 1–25% of the time; regularly = 25–50% of the time; often = 50–75%; very often =  ≥ 75% of the time)

During both the first and the second lockdown, 30–40% of the teachers used a digital environment to collaborate and assign tasks very often according to the school principals. Similarly, about one third of the teachers used digital coursebooks for reading very often during both lockdowns. In addition, results showed that about a quarter of the teachers never used a digital environment during the first lockdown. In the second lockdown, however, only one in eight teachers never used a digital environment. Similarly, about one fifth of the teachers did not provide online reading instruction during the first lockdown, whereas this was true for only one in ten teachers in the second lockdown.

Results furthermore showed that about one third of the school principals indicated for both the first and the second lockdown that students used digital software to practice reading very often, whereas about a quarter of the students never used digital software to practice reading according to the school principals.

Table 5 shows the results regarding monitoring of reading achievement during the first lockdown as well as during the second lockdown. About two-third of the teachers never used analyses of digital test results in their reading instruction during the first and second lockdown and over 70% of the teachers never used (formative) digital tests to monitor students’ reading achievement during both lockdowns. In addition, results showed that about half of the teachers never used digital coursebooks or (other) digital dashboards to monitor students’ reading achievement during both the first and second lockdowns. The proportion of teachers that analysed information from (other) digital dashboards to monitor students’ reading achievement very often was about a quarter during the first lockdown and about one out of eight during the second lockdown.

**Table 5:** Teachers’ monitoring of reading achievement practices during Covid19 lockdowns

	First Covid19 lockdown (March 2020–May 2020) n schools = 35					Second Covid19 lockdown (December 2020–February 2021) n school = 64				
	n (%) never	n (%) occasionally	n (%) regularly	n (%) often	n (%) very often	n (%) never	n (%) occasionally	n (%) regularly	n (%) often	n (%) very often
*Digitalization*										
Teachers use analyses of digital test results in their reading instruction	21 (60.0%)	4 (11.4%)	4 (11.4%)	2 (5.7%)	4 (11.4%)	44 (68.8%)	5 (7.8%)	4 (6.3%)	4 (6.3%)	7 (10.9%)
Teachers use results from digital coursebooks to monitor students’ reading achievement	16 (45.7%)	7 (20.0%)	6 (17.1%)	4 (11.4%)	2 (5.7%)	31 (48.4%)	9 (14.1%)	10 (15.6%)	9 (14.1%)	5 (7.8%)
Teachers use (formative) digital tests to monitor students’ reading achievement	25 (71.4%)	3 (8.6%)	3 (8.6%)	3 (8.6%)	1 (2.9%)	45 (70.3%)	11 (17.2%)	1 (1.6%)	3 (4.7%)	4 (6.3%)
Teachers analyse information from (other) digital dashboards to monitor students’ reading achievement	18 (51.4%)	4 (11.4%)	2 (5.7%)	3 (8.6%)	8 (22.9%)	33 (51.6%)	7 (10.9%)	9 (14.1%)	7 (10.9)	8 (12.5%)

Never = 0% of the time; occasionally = 1–25% of the time; regularly = 25–50% of the time; often = 50–75%; very often =  ≥ 75% of the time)

Next, results about the collaboration with parents were analyzed. About two-third (n = 21, 60.0%) of the school principals indicated that the collaboration with parents was a success factor and about one-third (n = 11, 31.4%) of the school principals specified the collaboration with parents as a difficulty during the first Covid-19 lockdown. For the second lockdown, the collaboration with parents was indicated as a success factor by about 80% of the school principals (n = 51, 79.7%) and as a difficulty by about 30% of the school principals (n = 19, 29.7%). Some school principals (n = 6 for the first lockdown and n = 13 for the second lockdown) indicated the collaboration with parents both as a success factor and a difficulty.

### Successful school practices during lockdowns

**Fig. 3:**
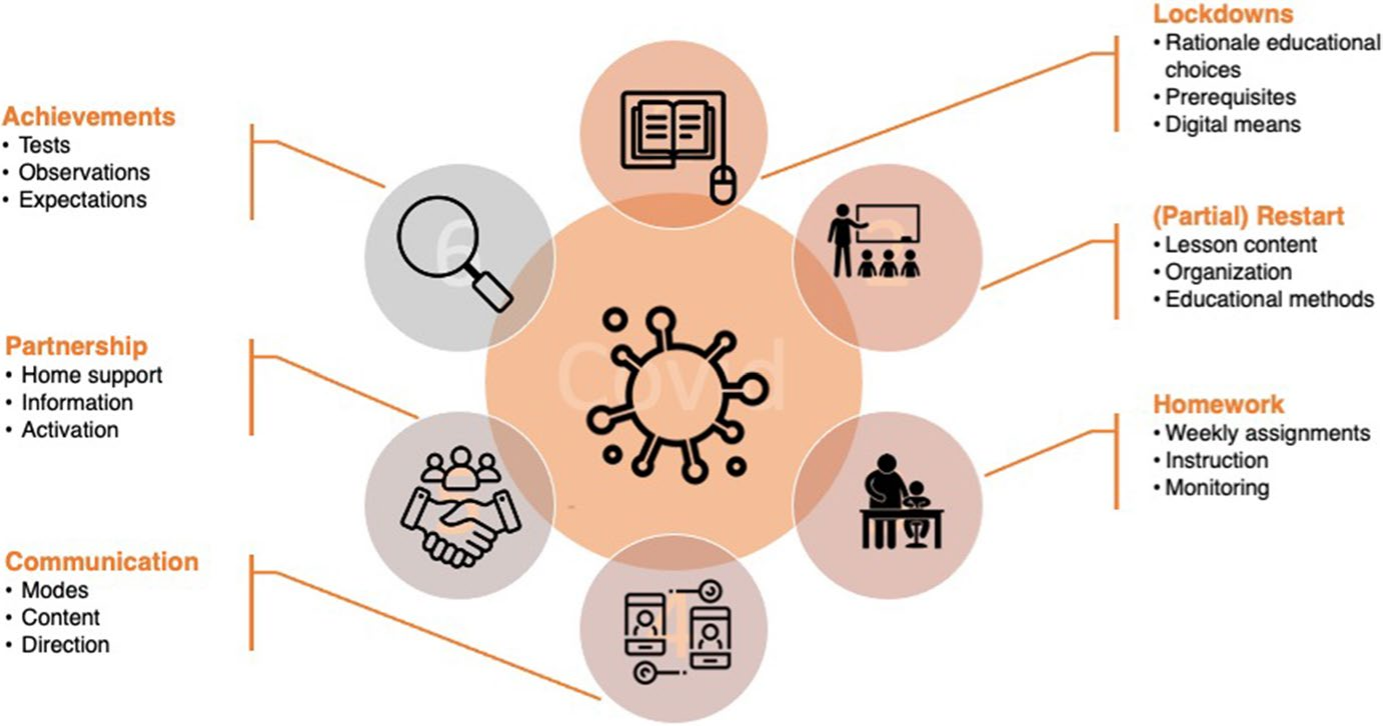
Conceptual model of success factors in lockdown and post lockdown education in resilient low SES schools

The interviews with five resilient (high performing) low-SES schools resulted in a conceptual model of protective actions during the lockdowns and restart of education (Fig. 3).

The rationale for educational choices made by these schools during the lockdowns were associated with the teachers’ concerns of overloading parents in providing home-schooling support for their children and burdening the students. Teachers felt supported by school management in the ways in which teaching and curriculum was tailored to the situation. During the first lockdown, the prerequisites for online education were often not present (devices children had access to, digital skills of both teachers and students), but in several schools, digital means were implemented to provide online classes during the first lockdown, increasingly. Although some teachers state that searching for digital content and preparing classes was time consuming and complex, digital exercises and instructional videos (from educational television shows or teacher-made) were available and used in online education. In addition, some schools gave students opportunities to borrow books from the school library to promote reading at home. In these lockdown time frames, continuity in contact with the students was a priority for the schools, to ensure that the wellbeing of the students was not under pressure and that homework activities were done according to schedule. Some schools especially focussed on core subjects (language, spelling, reading and math) and repetition of previously covered lesson content, whereas others continued the regular program. When reflecting on the second lockdown, teachers expressed more self-efficacy in organising online education. Several schools were able to provide laptops or other devices for children to take home, which meant an improvement in the digital preconditions. Many teachers recount to have benefited from the insights gained from the first lockdown period.

In the period of partial restart of onsite education, some schools opted for halved classes to be present at school (one half during the mornings, the other half during the afternoons), whereas other schools made the classes taking turns in coming to school (for example, grade 4–6 on Mondays and Thursdays, other grades on Tuesdays and Fridays). In-class time was mostly reserved for (small) group instruction or individual support; during the remainder of the week, the students processed the lesson materials independently at home using exercises that were provided by the teacher or a digital tutoring system/program.

Homework usually took the form of weekly assignments on group level; some schools also designed weekly tasks for individual students. When addressing reading comprehension in these weekly tasks, in many cases texts with assignments or questions were distributed. Checking of homework was often done by the teacher, but parents and students themselves were also encouraged to see if the homework was done correctly, using review templates. When students failed to hand in their homework on time, inquiries were made to the parents, but overall, the degree to which the assignments were made was satisfactory.

A large part of communication with parents that teachers initiated consisted of offering a helping hand or a sympathetic ear, addressing the concerns and problems parents were experiencing with the home-schooling situation, and inquiring about the well-being of the students. Teachers regularly asked questions about the achieved successes in doing homework and provided explanatory notes on the assignments the students had to complete. Digital means and telephone calls were used to contact the families, but in some cases, there was live contact, for instance when teachers delivered educational materials to the students’ homes.

Teachers made an effort to preserve effective educational partnerships with parents. Several actions were taken to inform parents on the role they could play in their child’s learning behaviour in general, and in reading development in particular. According to the teachers’ observations, parent involvement in children’s homework was successful in many cases. Yet, all teachers we interviewed mentioned that one or two families in their class had limited success in supporting their child’s educational needs. In these cases, the emergency classes that were set up at the school then could offer a solution.

The schools we interviewed varied on the aspect of monitoring student achievements. Some schools followed the regular testing protocol from the general student tracking system or method-related tests. Other schools, however, deviated from their testing calendar, because they thought it would put too much of a burden on the students, and testing in the online situation seemed impractical. Assessments in the post-lockdown periods were done to gain more insight into potential delays.

## Discussion

The first aim of the current study was to investigate development in reading comprehension performance of schools with students from low socio-economic or migrant backgrounds versus schools with students from high socio-economic backgrounds during and after school closure due to Covid-19. The second aim was to explore protective factors against negative effects, by pinpointing successful practices of resilient schools at the time of school closures. We found negative effects of the lockdowns, but not related to SES, and showed that schools were better prepared during the second lockdown. The use of digital materials and establishment of partnerships with parents were identified as promising protective factors.

The first research question focused on the impact of the Covid-19 lockdowns on the development of reading comprehension, and the role of school level SES. The results partially confirm the first hypothesis. We found that students in lower SES-school made less progress over time than students in higher-SES schools, and that the lockdowns had a negative effect on reading comprehension development across school-level SES. The fact that students in lower-SES schools make less progress in reading comprehension is in line with the pre Covid-19 literature (e.g., Caldas & Bankston, 1997; Kieffer, 2012). Contrary to our first hypothesis, we did not find that school-level SES related to specific lower development during the lockdowns. The faucet theory (Entwisle et al., 2001) may explain why these effects are different from those found due to summer holidays (as reviewed by Cooper et al., 1996). While during summer holidays, school resources are not available, which is especially negative for low SES-families, this was not the case during the lockdowns. Schools were -in a way- guiding the home environment, and as such attenuating SES-effects. Other studies on the effects of Covid-19, however, did find SES-effects (see Hammerstein et al., 2021), but Betthäuser et al. (2022) pointed out that in the 99 studies they collected on Covid-19 reading inequalities from March 2020 to October 2021, 71% showed an increase in inequality between students from different socio-economic backgrounds, while in 26% of the studies there was no change in inequality, and in 3% of the studies the inequality decreased. Our results do show that school-level SES has an impact on the development of reading comprehension, but this effect does not seem to be enlarged during the lockdowns, since already there was a divergent pattern before the lockdowns.

In addition, we are one of the first to show a partial recovery after the lockdowns, as students made more progress than the reference group after the lockdowns. Such effects are remarkable, considering that effects of interventions on standardized measures of reading comprehension are often small (the meta-analysis by Okkinga et al., 2018 reported an effect size of 0.186). It is positive to see that students are catching up, but it should also be noted that the effect of the lockdowns is still visible. We could not confirm our hypothesis that especially higher-SES schools would catch up. It is easy to assume that the catching up is due to more attention in school to reading comprehension (see e.g., Houtveen & Van de Grift, 2007).

Our second research question focused on the design of distance education. In line with our hypothesis, questionnaire-data revealed that schools were better prepared during the second lockdown, with teachers making more use of digital environments, digital coursebooks and digital dashboards, and providing more online reading instruction. In addition, collaboration with the parents seemed to have improved. The in-depth interviews helped to distinguish a number of compensatory measures of resilient low SES schools that may be relevant to minimize the negative impact of the two lockdown periods. We created a conceptual model (Fig. 3) to visualize these effects. They include the introduction and facilitation of online education, focusing teaching on what was feasible and urgent, providing homework assignments and books to read at home, applying alternative monitoring practices and investing in educational partnerships with parents using various communication modes and information formats. Engzell et al. (2021) noted the Netherlands to represent a best-case scenario because of the high technological standards. Indeed, it was observed that online education ("emergency remote teaching") made a spurt during the Covid-19 years (Drachsler et al., 2021).

The current study has several limitations that should be acknowledged. The first is that we did not include a control group of the same schools from previous years. We compared the current group to a reference group, but this group does not differentiate on SES. In line with this point, we could not directly compare the effect of the lockdowns to a control group that did not experience lockdowns, as the pandemic situation made such (ideal) design impossible. Second, while our sample is representative for the Netherlands, these results cannot be transferred to other countries. Comparable studies in other countries are needed to further understand the effect of the lockdowns globally. Finally, we interviewed resilient low-SES schools, but did not study what schools did to catch up after the lockdowns, and if this was at the cost of other school subjects or social aspects. It also would have been interesting to interview low-SES schools that did not perform well during lockdowns, in order to compare differences and identify barriers in dealing with school closures and online education. The results of the interviews, however, are of relevance to the field, as they may help other schools to organize their education, not only during a lockdown which we all hope to never experience again, but also in general education.

In the current study, we followed the reading comprehension development of 2202 students from 69 different schools, before, during and after two Covid-19 lockdowns. We conclude that these lockdowns have had a cumulative negative effect on the development of reading education, but that students partially catch up in the months after the second lockdown. Overall, the SES-level of the schools has a negative impact on the development of reading comprehension over time, but not especially more so during lockdowns. Digital means and partnership with parents might be protective factors to attenuate the negative effects of emergency remote teaching.
